# Health behaviors and metabolic risk factors are associated with dyslipidemia in ethnic Miao Chinese adults: the China multi-ethnic cohort study

**DOI:** 10.1186/s12889-021-10871-0

**Published:** 2021-05-03

**Authors:** Fang Nie, Ziyun Wang, Qibing Zeng, Han Guan, Jingyuan Yang, Peng Luo, Lunwei Du, Junhua Wang, Feng Hong

**Affiliations:** 1 School of Public Health, The Key Laboratory Of Environmental Pollution Monitoring and Disease Control, Ministry of Education, Guizhou Medical University, Guiyang, 550025 China; 2 School of Public Health, Guizhou Medical University, Guiyang, 550025 China

**Keywords:** Dyslipidemia, Cardiovascular health, Health behaviors, Metabolic risk factors, Minorities

## Abstract

**Background:**

Cardiovascular risk factors in Chinese ethnic minority groups are rarely reported.

**Objective:**

To quantify the cardiovascular risk factors in Miao Chinese adults and to examine the association of health behaviors and metabolic risk factors with dyslipidemia.

**Methods:**

A cross-sectional analysis was conducted using baseline data from the China Multi-Ethnic Cohort (CMEC) study. A representative sample of 5559 Miao participants aged 30 to 79 years were surveyed and given physical and laboratory exams. The proportion of behavioral and metabolic risk factors were described in ethnic Miao adults. Logistic regression was utilized to evaluate the odds ratio (OR) and 95% confidence interval (CI) of the association between health behaviors and metabolic risk factors with dyslipidemia.

**Results:**

In Miao Chinese adults, the prevalence of dyslipidemia was 32.8%. After multivariate adjustment, subjects with poor waist-to-hip ratio (WHR), body mass index (BMI), fasting blood glucose (FBG) and blood pressure (BP) were more likely to have higher risk of triglycerides (TG) abnormality, regardless of gender and age. Furthermore, the strongly association was detected between poor WHR and low density lipoprotein cholesterol (LDL-C) abnormality (adjusted OR = 5.24, 95%CI: 2.42–11.34) in the older subgroup (≥ 60 years). Males who current smoking were an independent risk factor only for high density lipoprotein cholesterol (HDL-C) abnormality (adjusted OR = 1.44, 95%CI: 1.05–1.99). However, in the subgroup age, current smoker were at greater risk of high TG and low HDL-C. Males with regular drinking were less likely to be high LDL-C (adjusted OR = 0.51, 95%CI: 0.32–0.81).

**Conclusions:**

The present findings indicated that Miao adults with metabolic risk factors were at greater risk of dyslipidemia.

## Background

Cardiovascular disease (CVD) is a public health problem with high morbidity and mortality and accounts for over 40% of deaths from all causes [1]. Dyslipidemia is a well-established risk factor for CVD [2]. Elevated total cholesterol (TC) and low density lipoprotein cholesterol (LDL-C) abnormality are generally considered to be independent risk factors for atherosclerosis [3]. Studies showed that for each 1 mmol/L decrease in LDL-C, this risk of cardiovascular event is reduced by 21% [4]. In clinical practice, these metabolic signs are the most commonly used indicators of CVD risk [5, 6].

Epidemiological data show that 64.4% of the Chinese population has at least one type of dyslipidemia [7]. The high prevalence of dyslipidemia is related to a dramatic shift to an unhealthy lifestyle [8]. Prior study has reported that the lipid metabolism is closely related to lifestyle factors [9, 10]. Multiple healthy lifestyle may be as effective as drug therapy at preventing dyslipidemia, and these lifestyle factors are better managed and moderated [11]. Therefore, the increased prevalence of dyslipidemia is likely attributable to lifestyle risk factors.

In Guizhou province, the Miao ethnic group accounts for 42.1% of all Miao people according to the sixth census [12]. The Miao people have deep historic roots and still maintain old customs and a unique lifestyle that are distinct from the majority of Han Chinese [13]. Although dyslipidemia is well documented by studies in the Han people, the evidence poorly understood in ethnic Chinese minority groups and the practice of dyslipidemia management is still limited. Consequently, to better understand the association between health behaviors and metabolic risk factors with dyslipidemia, we conducted an analysis using baseline data from the China Multi-Ethnic Cohort (CMEC) study.

## Materials and methods

### Study participants

The CMEC study is a large-scale prospective cohort study based on community population in five provinces of southwest China, Guizhou, Yunnan, Sichuan, Chongqing and Tibet. From July 2018 to April 2019, 99,556 members aged 30 to 79 years old were recruited. Further details are available elsewhere [14].

Multi-stage stratified random sampling was used to select the study participants. The Miao and Dong autonomous prefecture of Qiandongnan, and the Bouyei and Miao autonomous prefecture of Qiannan were selected based on the characteristics of ethnic minority groups in Guizhou. Next, from these prefecture, Kaili City, Liping County, and Libo County were selected as secondary sampling units.

### Questionnaire and physical examinations

More detailed information on questionnaire and medical examinations are published elsewhere [14]. In brief, information on health behaviors was collected by questionnaire in face-to-face interviews with trained medical student. Behavioral data included: smoking status, alcohol consumption status, average sleep duration, and physical activity (PA). PA was calculated as the total of the metabolic equivalent of each task in hours per day.

Blood sample was collected by professional nurses from participants after a mandatory 8-h fast. Samples were sent to the JinYu Medical Laboratory Center of Guizhou Province for analysis of TC, LDL-C, high density lipoprotein cholesterol (HDL-C), triglycerides (TG), and fasting blood glucose (FBG).

BP was recorded as the average of three measurements taken at 5-min intervals using an electronic sphygmomanometer. WHR was calculated from the ratio of waist circumference to hip circumference. Waist and hip circumference were measured using soft tape. BMI was defined as weight in kilograms divided by the square of the height in meters (kg/m^2^).

Participant responses were categorized as Ideal, Intermediate, or Poor based on definitions listed in Table 1. The categories were defined by guidelines form the American Heart Association combined with characteristics specific to Chinese people [15, 16].

**Table 1:** Classification of health behaviors and metabolic risk factors

Variables	Ideal	Intermediate	Poor
**Smoking status**	Non-smoker	Previous smoker (smoking cessation ≥1 year)	Current smoker
**Alcohol consumption**	Non-drinker	Occasional drinker	Regular drinker (weekly or monthly drinking)
**Sleep duration, hours**	7 ~ 8	5–6 or 9–10	< 5 or > 10
**BMI, kg/m**^**2**^	< 24	24–27.9	≥ 28
**WHR (Male)**	< 0.90	0.90–0.94	≥ 0.95
**(Female)**	< 0.85	0.85–0.89	≥ 0.90
**BP, mmHg**	Non-drug therapy < 120/80	120–139/80–89 or < 140/90 if taking an antihypertensive	≥ 140/90
**FBG, mmol/L**	Non-drug therapy < 5.6	5.6–6.9 or < 5.6 if taking diabetes drugs	≥ 7.0
**PA, (MET-hour/day)**	≥ P50	P25-P50	< P25

*BMI* Body mass index, *FBG* Fasting blood glucose, *WHR* Waist-to-hip ratio, *MET* Metabolic equivalent task, *BP* Blood pressure, *PA* Physical activity

### Dyslipidemia outcomes

According to published guidelines for Chinese adults [17], dyslipidemia was defined the presence of any of these four indicators: TC ≥ 6.22 mmol/L, TG ≥ 2.26 mmol/L, LDL-C ≥ 4.14 mmol/L, or HDL-C <  1.04 mmol/L.

### Statistical analysis

Categorical variables were expressed as n (%). The demographic characteristics between different groups were compared using χ^2^ test for categorical variables. The distribution of health behaviors and metabolic risk factors were summarized by gender, age and place of residence. The prevalence of dyslipidemia was stratified by gender. We also described the prevalence by each component of lipid. The age-standardized prevalence of dyslipidemia was calculated corresponding to the sixth census of Guizhou in 2010.

Univariate and multivariate logistic regression was used to evaluate the association between health behaviors and metabolic risk factors with dyslipidemia components, stratified by gender. High TC, high TG, high LDL-C and low HDL-C were used as the dependent variables. The regression model was adjusted for age, area of residence, education, occupation, marital status and family income as defined in Table 2. The analysis was also stratified to subjects < 60 and ≥ 60 years old.

**Table 2:** Characteristics of participants

Variables	Total (***n*** = 5032)	Male (***n*** = 1845)	Female (***n*** = 3187)	***P -*** value^*^
**Age (years, %)**				< 0.001
**30–39**	862 (17.2)	277 (15.0)	585 (18.4)	
**40–49**	1478 (29.4)	500 (27.1)	978 (30.6)	
**50–59**	1396 (27.7)	490 (26.6)	906 (28.4)	
**60–69**	893 (17.7)	397 (21.5)	496 (15.6)	
**70–79**	403 (8.0)	181 (9.8)	222 (7.0)	
**Area of residence (%)**				0.056
**Rural**	3584 (71.2)	1289 (69.9)	2295 (72.0)	
**Urban**	1448 (28.8)	556 (30.1)	892 (28.0)	
**Educational level (%)**				< 0.001
**No formal school**	2190 (43.5)	540 (29.2)	1650 (51.7)	
**Primary school**	687 (13.7)	330 (17.9)	357 (11.2)	
**Middle school**	1004 (20.0)	400 (21.7)	604 (19.0)	
**High school or college**	884 (17.5)	418 (22.7)	466 (14.6)	
**University or above**	267 (5.3)	157 (8.5)	110 (3.5)	
**Occupation (%)**				< 0.001
**Farmers**	1767 (35.1)	725 (39.3)	1042 (32.7)	
**Workers**	400 (7.9)	186 (10.1)	214 (6.7)	
**Administrators**	165 (3.3)	95 (5.1)	70 (2.2)	
**Specialists**	441 (8.8)	237 (12.8)	204 (6.4)	
**Other occupations**	2259 (44.9)	602 (32.7)	1657 (52.0)	
**Marital status (%)**				< 0.001
**Married or cohabiting**	4367 (86.8)	1644 (89.1)	2723 (85.4)	
**Separated or divorced**	183 (3.6)	62 (3.4)	121 (3.8)	
**Widowed**	427 (8.5)	87 (4.7)	340 (10.7)	
**Never married**	55 (1.1)	52 (2.8)	3 (0.1)	
**Family income (yuan/year, %)**				
**< 20,000**	2101 (41.7)	772 (41.8)	1329 (41.7)	0.783
**20,000-59,999**	1650 (32.8)	591 (32.0)	1059 (33.2)	
**60,000-99,999**	763 (15.2)	289 (15.7)	474 (14.9)	
**≥ 100,000**	518 (10.3)	193 (10.5)	325 (10.2)	

Data was presented as n (%)

^*^Chi-square test

All statistical analysis were performed using IBM SPSS statistics for windows, version 22.0 (IBM Corp., Armonk, NY, USA) and R 4.0.2 (R Core Team). Statistical tests were two-tailed and statistical significance was set at *P* ≤ 0.05.

## Results

### Characteristics of participants

Of the 5559 participants entering this study, 5032 (90.5%) completed questionnaire and physical examination. Women comprised 63.3% of the sample (*n* = 3187). Participants were excluded if they had missing data on those variables fasting time < 8 h (*n* = 8), lacked of blood samples (*n* = 483), or were using hyperlipidemic medication (*n* = 36).

The demographic characteristics are presented in Table 2. The average age of the participants was (51.8 ± 11.7) years. Men were generally older, had more education compared to women. Most men worked as farmers while women worked in other occupations.

### Distribution of health behaviors and metabolic risk factors

Table 3 summarizes the distribution of health behaviors and metabolic risk factors by gender, age and area of residence. Overall, the proportion of subjects with ideal WHR (32.9%), BMI (40.2%) and BP (33.4%) were relatively low. However, there were high percentages of both non-smokers (78.1%) and of those with ideal FBG (73.5%) levels. Current smokers and regular drinkers were more likely to be men, to be older than 60, and living in rural areas. About half of all women had a poor WHR (50.2%). But among women, nearly all were non-smokers (98.9%), a majority were non-drinkers (53.7%), and over three-quarter had ideal FBG (77.7%).

**Table 3:** Distribution of health behaviors and metabolic risk factors by gender, age and area of residence

Variables	Total	Gender		Age (years)		Area of residence	
		Male (***n*** = 1845)	Female (***n*** = 3187)	< 60 (***n*** = 3736)	≥ 60 (***n*** = 1296)	Rural (***n*** = 3584)	Urban (***n*** = 1448)
**Smoking status (%)**							
**Ideal**	3933 (78.1)	780 (42.1)	3153 (98.9)	2964 (79.3)	969 (74.8)	2799 (78.1)	1134 (78.3)
**Intermediate**	190 (3.8)	184 (10.1)	6 (0.2)	121 (3.2)	69 (5.3)	122 (3.4)	68 (4.7)
**Poor**	909 (18.1)	881 (47.8)	28 (0.9)	651 (17.5)	258 (19.9)	663 (18.5)	246 (17.0)
**Alcohol consumption (%)**							
**Ideal**	2251 (44.7)	541 (29.3)	1710 (53.7)	1514 (40.6)	737 (56.9)	1662 (46.4)	589 (40.7)
**Intermediate**	1881 (37.4)	709 (38.5)	1172 (36.7)	1559 (41.7)	322 (24.8)	1273 (35.5)	608 (42.0)
**Poor**	900 (17.9)	595 (32.2)	305 (9.6)	663 (17.7)	237 (18.3)	649 (18.1)	251 (17.3)
**PA (MET- hour/day, %)**							
**Ideal**	2516 (50.0)	948 (51.4)	1568 (49.2)	2056 (55.0)	460 (35.5)	1985 (55.4)	531 (36.7)
**Intermediate**	1258 (25.0)	445 (24.1)	813 (25.5)	926 (24.8)	332 (25.6)	809 (22.6)	449 (31.0)
**Poor**	1258 (25.0)	452 (24.5)	806 (25.3)	754 (20.2)	504 (38.9)	790 (22.0)	468 (32.3)
**WHR (%)**							
**Ideal**	1654 (32.9)	788 (42.7)	866 (27.1)	1267 (33.9)	387 (29.8)	1197 (33.4)	457 (31.6)
**Intermediate**	1184 (23.5)	462 (25.1)	722 (22.7)	906 (24.3)	278 (21.5)	812 (22.7)	372 (25.7)
**Poor**	2194 (43.6)	595 (32.2)	1599 (50.2)	1563 (41.8)	631 (48.7)	1575 (43.9)	619 (42.7)
**BMI (kg/m**^**2**^**, %)**							
**Ideal**	2021 (40.2)	782 (42.4)	1239 (38.9)	1429 (38.2)	592 (45.7)	1482 (41.4)	539 (37.2)
**Intermediate**	2093 (41.6)	776 (42.0)	1317 (41.3)	1605 (43.0)	488 (37.6)	1449 (40.4)	644 (44.5)
**Poor**	918 (18.2)	287 (15.6)	631 (19.8)	702 (18.8)	216 (16.7)	653 (18.2)	265 (18.3)
**FBG (mmol/L, %)**							
**Ideal**	3701 (73.5)	1226 (66.5)	2475 (77.7)	2843 (76.1)	858 (66.2)	2704 (75.4)	997 (68.9)
**Intermediate**	1035 (20.6)	458 (24.8)	577 (18.1)	714 (19.1)	321 (24.8)	706 (19.7)	329 (22.7)
**Poor**	296 (5.9)	161 (8.7)	135 (4.2)	179 (4.8)	117 (9.0)	174 (4.9)	122 (8.4)
**BP (mmHg, %)**							
**Ideal**	1683 (33.4)	391 (21.2)	1292 (40.5)	1473 (39.4)	210 (16.2)	1200 (33.5)	483 (33.3)
**Intermediate**	1862 (37.0)	741 (40.2)	1121 (35.2)	1347 (36.1)	515 (39.7)	1331 (37.1)	531 (36.7)
**Poor**	1478 (29.6)	713 (38.6)	774 (24.3)	916 (24.5)	571 (44.1)	1053 (29.4)	434 (30.0)
**Sleep duration (hour, %)**							
**Ideal**	2579 (51.3)	904 (49.0)	1675 (52.6)	2041 (54.6)	538 (41.5)	298 (8.3)	75 (5.2)
**Intermediate**	2080 (41.3)	776 (42.1)	1304 (40.9)	1505 (40.3)	575 (44.4)	1540 (43.0)	540 (37.3)
**Poor**	373 (7.4)	165 (8.9)	208 (6.5)	190 (5.1)	183 (14.1)	1746 (48.7)	833 (57.5)

Data was presented as n (%)

*BMI* Body mass index, *FBG* Fasting blood glucose, *WHR* Waist-to-hip ratio, *BP* Blood pressure, *PA* Physical activity, *MET* Metabolic equivalent task

### Prevalence of dyslipidemia

The prevalence of dyslipidemia in this cohort of Miao participants was 32.8%, which was age-standardized was 23.3% (Table 4). Dyslipidemia was more likely to be present in men than women (42.2% vs 27.5%, *P* <  0.001). In both genders, there was more dyslipidemia in participants who had poor WHR, BMI, FBG, and BP (each *P* <  0.001). Table 5 presents the prevalence of dyslipidemia components. The prevalence of high TC, TG, LDL-C and low HDL-C was 10.4%, 21.8%, 7.7% and 7.4%, respectively. Again, participants with poor WHR, BMI, FBG and BP were more likely to have prevalence of dyslipidemia components.

**Table 4:** Adjusted odds ratio (95% CI) of dyslipidemia prevalence

Variables	Male (***n*** = 778)		Female (***n*** = 875)	
	Dyslipidemia	OR (95%CI)^**a**^	Dyslipidemia	OR (95%CI)^**a**^
**Smoking status (%)**				
**Ideal**	326 (41.8)	1.00	869 (27.6)	1.00
**Intermediate**	73 (39.7)	0.64 (0.70–5.86)	1 (16.7)	0.73 (0.08–6.65)
**Poor**	379 (43.0)	0.67 (0.24–1.83)	5 (17.9)	0.69 (0.25–1.94)
**Alcohol consumption (%)**				
**Ideal**	190 (35.1)	1.00	498 (29.1)	1.00
**Intermediate**	331 (46.7)	0.90 (0.75–1.07)	290 (24.7)	0.89 (0.74–1.07)
**Poor**	257 (43.2)	1.07 (0.81–1.43)	87 (28.5)	1.09 (0.81–1.45)
**PA (MET-hour/day, %)**				
**Ideal**	393 (41.5)	1.00	380 (24.2)	1.00
**Intermediate**	202 (45.4)	1.23 (1.00–1.50)	234 (28.8)	1.21 (0.98–1.49)
**Poor**	183 (40.5)	1.27 (1.04–1.55)^*^	261 (32.4)	1.26 (1.02–1.57)^*^
**WHR (%)**				
**Ideal**	218 (27.7)	1.00	132 (15.2)	1.00
**Intermediate**	208 (45.0)	1.33 (1.02–1.72)^*^	172 (23.8)	1.28 (0.98–1.67)
**Poor**	352 (59.2)	1.92 (1.52–2.42)^*^	571 (35.7)	1.83 (1.44–2.33)^*^
**BMI (kg/m**^**2**^**, %)**				
**Ideal**	205 (26.2)	1.00	226 (18.2)	1.00
**Intermediate**	400 (51.5)	1.49 (1.22–1.82)^*^	396 (30.1)	1.49 (1.22–1.83)^*^
**Poor**	173 (60.3)	1.85 (1.46–2.35)^*^	253 (40.1)	1.87 (1.47–2.37)^*^
**FBG (mmol/L, %)**				
**Ideal**	449 (36.6)	1.00	576 (23.3)	1.00
**Intermediate**	223 (48.7)	1.53 (1.25–1.87)^*^	218 (37.8)	1.50 (1.22–1.85)^*^
**Poor**	106 (65.8)	3.16 (2.18–4.58)^*^	81 (60.0)	3.08 (2.12–4.48)^*^
**BP (mmHg, %)**				
**Ideal**	122 (31.2)	1.00	238 (18.4)	1.00
**Intermediate**	308 (41.6)	1.49 (1.22–1.81)^*^	338 (30.2)	1.45 (1.18–1.78)^*^
**Poor**	348 (48.8)	1.87 (1.50–2.32)^*^	299 (38.6)	1.81 (1.44–2.28)^*^
**Sleep duration (hour, %)**				
**Ideal**	379 (41.9)	1.00	444 (26.5)	1.00
**Intermediate**	338 (43.6)	0.93 (0.79–1.11)	359 (27.5)	0.94 (0.79–1.12)
**Poor**	61 (37.0)	1.08 (0.78–1.49)	72 (34.6)	1.11 (0.79–1.54)

*BMI* Body mass index, *FBG* Fasting blood glucose, *WHR* Waist-to-hip ratio, *BP* Blood pressure, *PA* Physical activity, *OR* Odds ratio, *CI* Confidence interval, *MET* Metabolic equivalent task

^*^P < 0.001

^a^Adjusted for demographic characteristics, including age, area of residence, education, occupation, marital status and family income

**Table 5:** Adjusted odds ratio (95% CI) of dyslipidemia components prevalence

Variables	TC (≥ 6.22 mmol/L)		TG (≥ 2.26 mmol/L)		LDL-C (≥ 4.14 mmol/L)		HDL-C (< 1.04 mmol/L)	
	n (%)	OR (95%CI)	n (%)	OR (95%CI)	n (%)	OR (95%CI)	n (%)	OR (95%CI)
**Smoking status (%)**								
**Ideal**	417 (10.6)	1.00	771 (19.6)	1.00	304 (7.7)	1.00	227 (5.8)	1.00
**Intermediate**	23 (12.1)	1.08 (0.68–1.72)	52 (27.4)	1.60 (1.12–2.30)^#^	12 (6.3)	0.78 (0.42–1.45)	20 (10.5)	2.30 (1.38–3.81)^#^
**Poor**	94 (10.3)	0.96 (0.74–1.25)	275 (30.3)	1.70 (1.40–2.06)^*^	69 (7.6)	1.15 (0.84–1.55)	124 (13.6)	2.86 (2.19–3.73)^*^
**Alcohol consumption (%)**								
**Ideal**	248 (11.0)	1.00	412 (18.3)	1.00	191 (8.5)	1.00	143 (6.4)	1.00
**Intermediate**	191 (10.2)	1.02 (0.83–1.27)	430 (22.9)	1.18 (0.99–1.39)	131 (7.0)	0.82 (0.64–1.04)	143 (7.6)	1.01 (0.78–1.30)
**Poor**	95 (10.6)	0.93 (0.71–1.22)	256 (28.4)	1.34 (1.09–1.65)^#^	63 (7.0)	0.75 (0.54–1.04)	85 (9.4)	1.02 (0.74–1.40)
**PA (MET-hour/day, %)**								
**Ideal**	241 (9.6)	1.00	513 (20.4)	1.00	172 (6.8)	1.00	156 (6.2)	1.00
**Intermediate**	136 (10.8)	1.03 (0.81–1.30)	302 (24.0)	1.13 (0.94–1.37)	99 (7.9)	0.96 (0.73–1.27)	115 (9.1)	1.45 (1.11–1.89)^*^
**Poor**	157 (12.5)	1.11 (0.87–1.41)	283 (22.5)	1.17 (0.98–1.40)	114 (9.1)	0.97 (0.74–1.29)	100 (7.9)	1.31 (0.98–1.76)
**WHR (%)**								
**Ideal**	110 (6.7)	1.00	196 (11.9)	1.00	58 (3.5)	1.00	68 (4.1)	1.00
**Intermediate**	107 (9.0)	1.07 (0.80–1.44)	270 (22.8)	1.63 (1.31–2.03)^*^	76 (6.4)	1.62 (1.13–2.33)^#^	74 (6.3)	1.38 (0.97–1.98)
**Poor**	317 (14.4)	1.58 (1.21–2.05)^*^	632 (28.8)	1.98 (1.61–2.43)^*^	251 (11.4)	2.81 (2.02–3.90)^*^	229 (10.4)	2.32 (1.68–3.20)^*^
**BMI (kg/m**^**2**^**, %)**								
**Ideal**	160 (7.9)	1.00	230 (11.4)	1.00	115 (5.7)	1.00	82 (4.1)	1.00
**Intermediate**	242 (11.6)	1.13 (0.89–1.42)	550 (26.3)	1.99 (1.65–2.39)^*^	174 (8.3)	1.00 (0.77–1.30)	185 (8.8)	1.81 (1.36–2.43)^*^
**Poor**	132 (14.4)	1.18 (0.89–1.55)	318 (34.6)	2.48 (1.99–3.10)^*^	96 (10.5)	1.01 (0.74–1.39)	104 (11.3)	2.10 (1.49–2.95)^*^
**FBG (mmol/L, %)**								
**Ideal**	321 (8.7)	1.00	645 (17.4)	1.00	234 (6.3)	1.00	224 (6.1)	1.00
**Intermediate**	144 (13.9)	1.30 (1.04–1.62)^#^	308 (29.8)	1.55 (1.31–1.84)^*^	112 (10.8)	1.37 (1.07–1.72)^#^	99 (9.6)	1.32 (1.01–1.72)^#^
**Poor**	69 (23.3)	2.08 (1.53–2.84)^*^	145 (49.0)	2.97 (2.27–3.88)^*^	39 (13.2)	1.39 (1.00–2.04)^#^	48 (16.2)	2.05 (1.42–2.97)^*^
**BP (mmHg, %)**								
**Ideal**	96 (5.7)	1.00	210 (12.5)	1.00	84 (5.0)	1.00	92 (5.5)	1.00
**Intermediate**	205 (11.0)	1.64 (1.26–2.12)^*^	424 (22.8)	1.67 (1.37–2.02)^*^	150 (8.1)	1.42 (1.06–1.90)^#^	153 (8.2)	1.25 (0.94–1.66)
**Poor**	233 (15.7)	2.12 (1.61–2.78)^*^	464 (31.2)	2.21 (1.80–2.72)^*^	151 (10.2)	1.58 (1.16–2.15)^#^	126 (8.5)	1.07 (0.77–1.47)
**Sleep duration (hour, %)**								
**Ideal**	267 (10.4)	1.00	544 (21.1)	1.00	189 (7.3)	1.00	179 (6.9)	1.00
**Intermediate**	220 (10.6)	0.92 (0.76–1.12)	464 (22.3)	1.07 (0.92–1.25)	153 (7.4)	0.95 (0.75–1.19)	165 (7.9)	1.15 (0.92–1.45)
**Poor**	47 (12.6)	1.00 (0.71–1.41)	90 (24.1)	1.22 (0.92–1.62)	43 (11.5)	1.49 (0.98–2.15)	27 (7.2)	1.05 (0.67–1.63)

Ideal group was used as reference, and non-dyslipidemia as control group. Adjusted for age, area of residence, education, occupation, marital status and family income

*BMI* Body mass index, *FBG* Fasting blood glucose, *WHR* Waist-to-hip ratio, *BP* Blood pressure, *PA* Physical activity, *MET* Metabolic equivalent task, *TC* Total cholesterol, *TG* Triglyceride, *LDL-C* Low-density lipoprotein cholesterol, *HDL-C* High-density lipoprotein cholesterol, *OR* Odds ratio, *CI* Confidence interval

^*^P < 0.001, ^#^ *P* < 0.05

### Association of health behaviors and metabolic risk factors with dyslipidemia

**Fig. 1:**
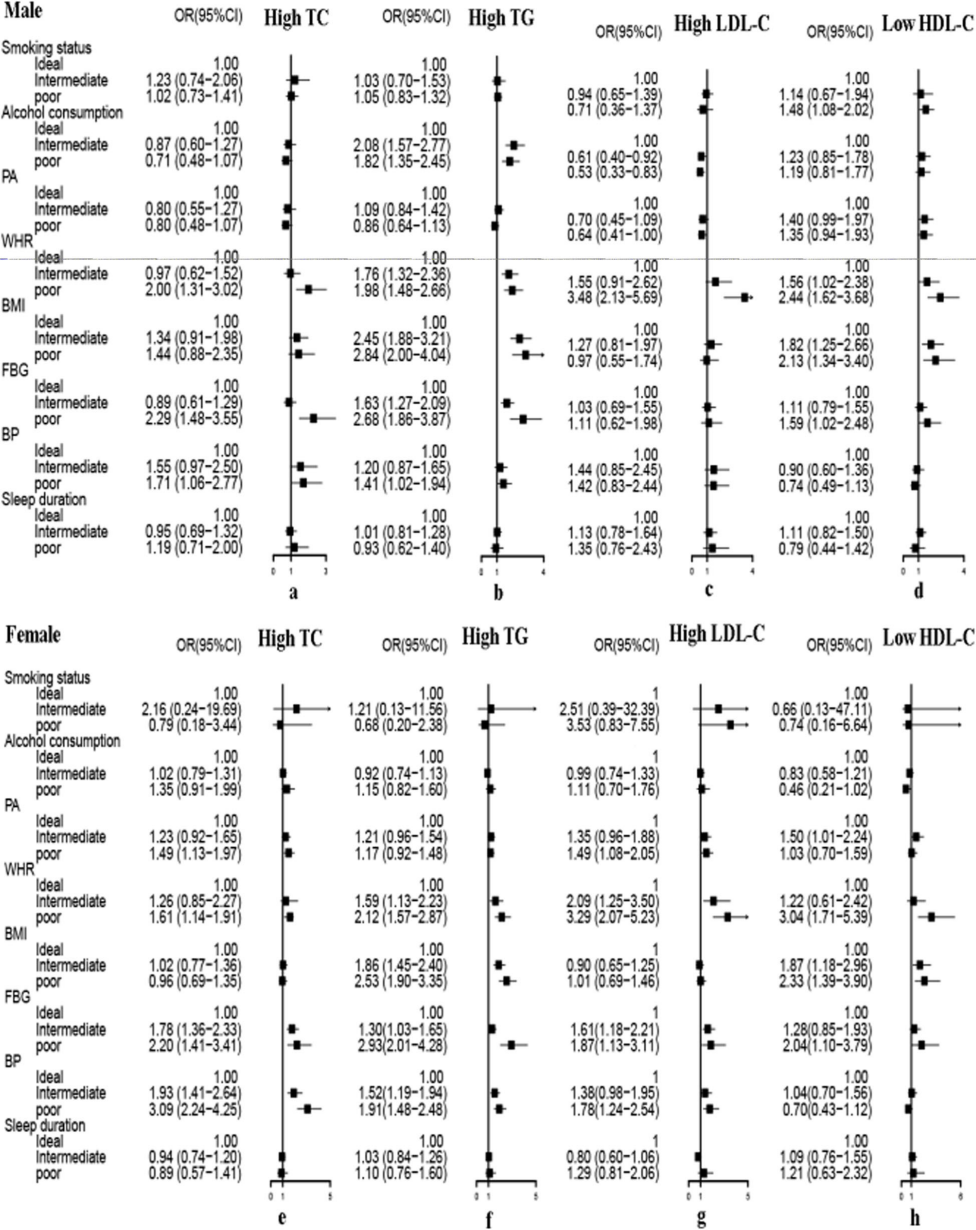
Association of health behaviors and metabolic risk factors with dyslipidemia components stratified by gender. No variables were adjusted in Figure 1. *BMI:* body mass index, *FBG:* fasting blood glucose, *WHR:* waist-to-hip ratio, *BP:* blood pressure, *PA:* physical activity, *OR:* odds ratio, *CI:* confidence interval, *TC:* total cholesterol, *TG:* triglycerides, *LDL-C:* low density lipoprotein cholesterol, *HDL-C:* high density lipoprotein cholesterol

**Fig. 2:**
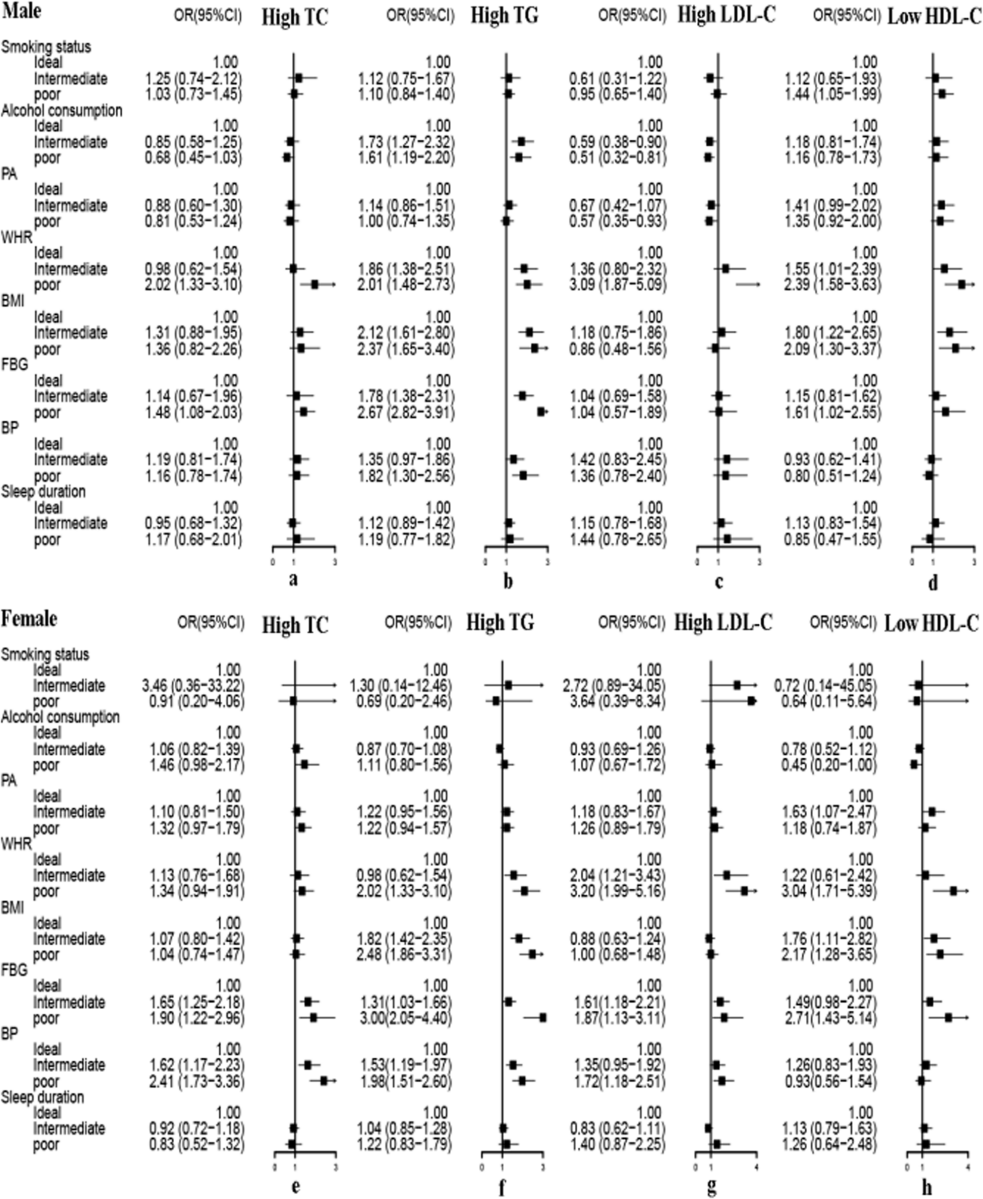
The association of health behaviors and metabolic risk factors with dyslipidemia components by gender after multivariate adjustment. Model was adjusted for age, area of residence, education, occupation, marital status and family income. *BMI:* body mass index, *FBG:* fasting blood glucose, *WHR:* waist-to-hip ratio, *BP:* blood pressure, *PA:* physical activity, *OR:* odds ratio, *CI:* confidence interval, *TC:* total cholesterol, *TG:* triglycerides, *LDL-C:* low density lipoprotein cholesterol, *HDL-C:* high density lipoprotein cholesterol

Figures 1 and 2 present the unadjusted and adjusted association of health behaviors and metabolic risk factors with dyslipidemia by gender, respectively. Figure 2 adjusted for age, residence, education, occupation, marital status and family income. High TG is likely to appear in subjects with poor BMI, WHR, BP and FBG. The odds of finding high lipid levels in subjects with poor WHR were high. The strongest associations were in subjects with poor WHR and high LDL-C (adjusted OR = 3.09 for men and adjusted OR = 3.20 for women, *P* <  0.001), low HDL-C (adjusted OR = 2.39 for men and adjusted OR = 3.04 for women, *P* <  0.001). The odds of having low HDL-C was the highest in poor FBG group in women (adjusted OR = 2.71, 95% CI: 1.43–5.14). Men who reported drinking regularly were more inclined to also have high TG (adjusted OR = 1.61, 95% CI: 1.19–2.20), but were less likely to have high LDL-C (adjusted OR = 0.51, 95% CI: 0.32–0.81). Males with current smoking were an independent risk factor only for low HDL-C (adjusted OR = 1.44, 95% CI: 1.05–1.99).

**Fig. 3:**
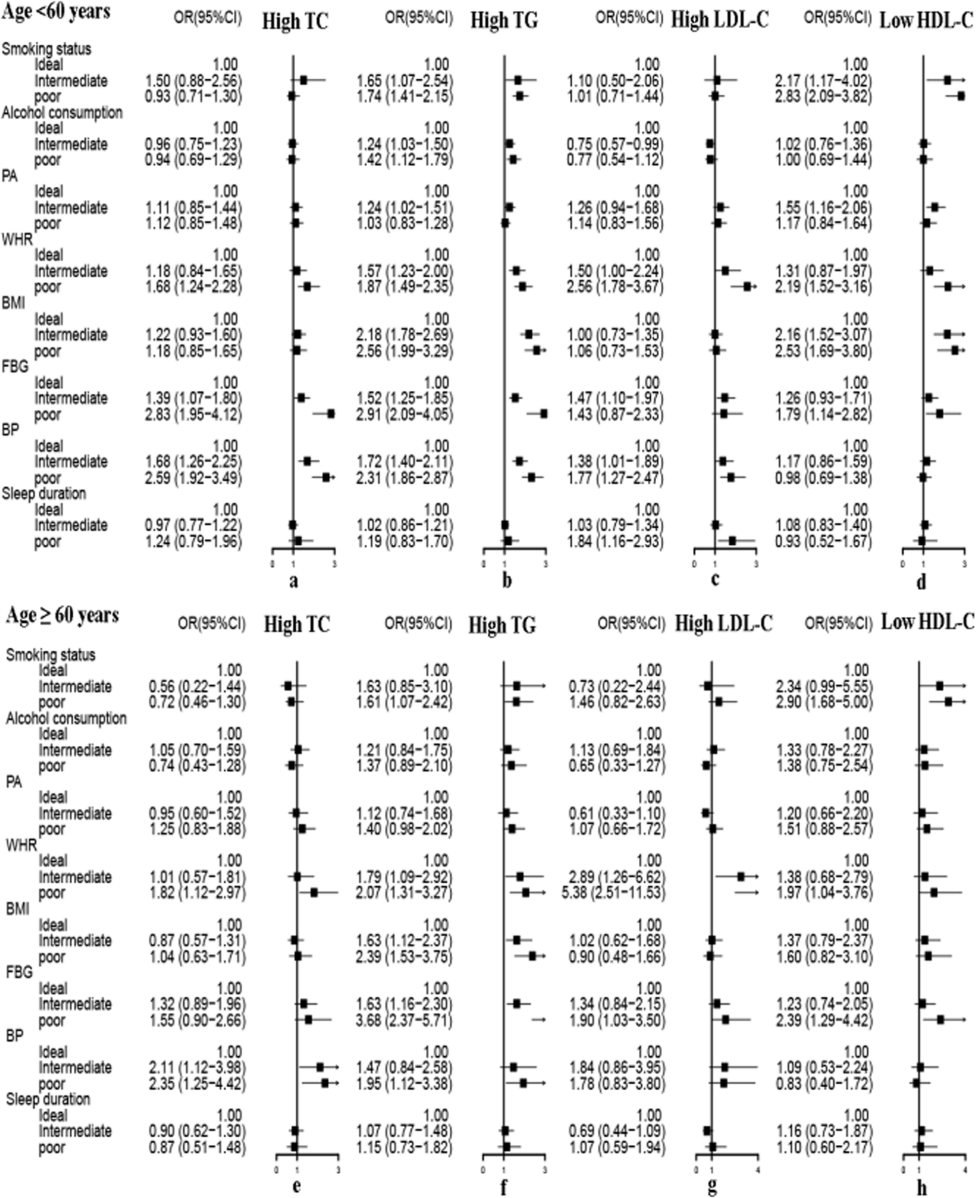
Association of health behaviors and metabolic risk factors with dyslipidemia components stratified by age. No variables were adjusted in Figure 3. *BMI:* body mass index, *FBG:* fasting blood glucose, *WHR:* waist-to-hip ratio, *BP:* blood pressure, *PA:* physical activity, *OR:* odds ratio, *CI:* confidence interval, *TC:* total cholesterol, *TG:* triglycerides, *LDL-C:* low density lipoprotein cholesterol, *HDL-C:* high density lipoprotein cholesterol

**Fig. 4:**
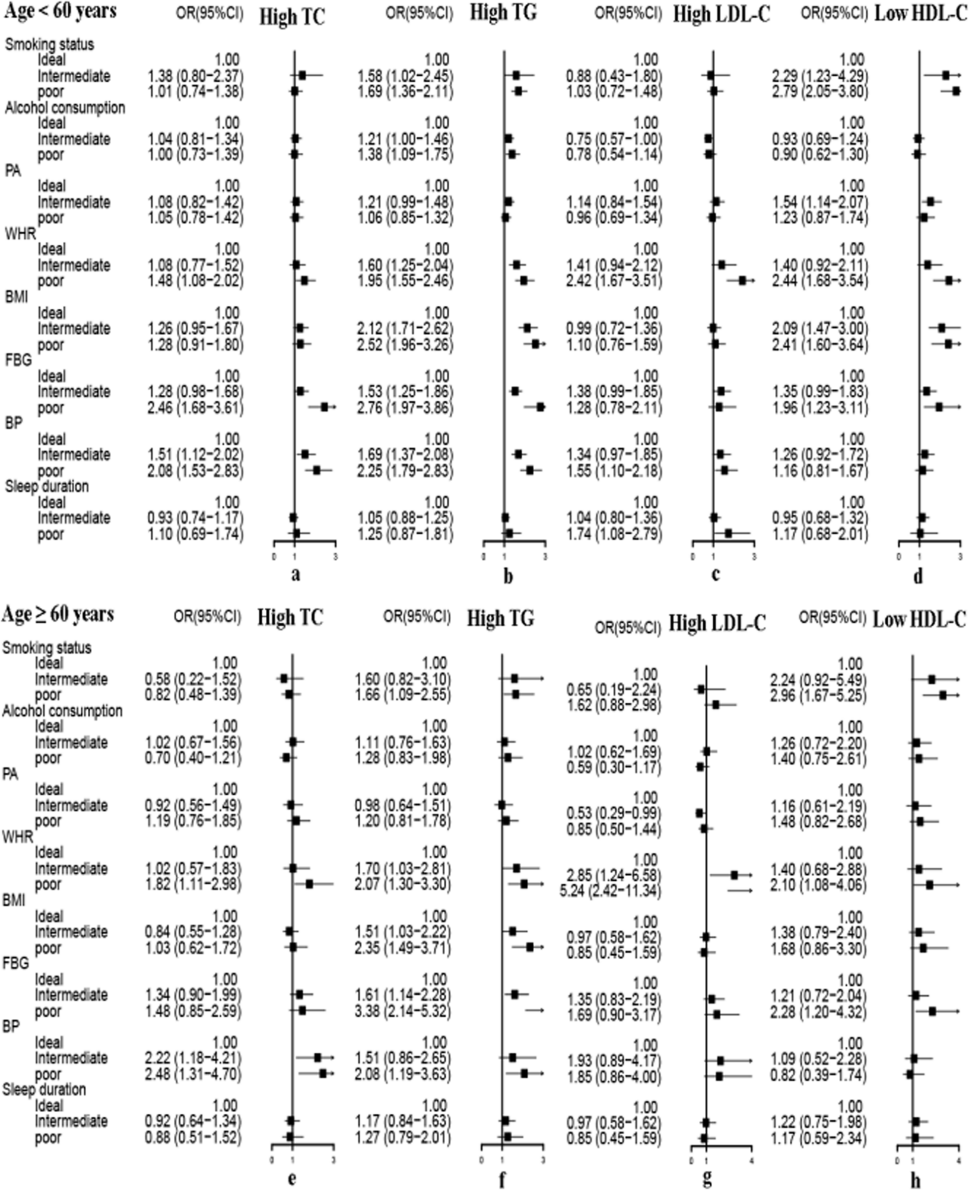
The association of health behaviors and metabolic risk factors with dyslipidemia components stratified by age after multivariate adjustment. Model was adjusted for age, area of residence, education, occupation, marital status and family income. *BMI:* body mass index, *FBG:* fasting blood glucose, *WHR:* waist-to-hip ratio, *BP:* blood pressure, *PA:* physical activity, *OR:* odds ratio, *CI:* confidence interval, *TC:* total cholesterol, *TG:* triglycerides, *LDL-C:* low density lipoprotein cholesterol, *HDL-C:* high density lipoprotein cholesterol

Figures 3 and 4, respectively, summarizes the unadjusted and adjusted association of health behaviors and metabolic risk factors with dyslipidemia by age. Stratification by age did not change the association between high TG and poor BMI, WHR, BP and FBG. In participants ≥60 years old, there was a strong association between poor WHR ang elevated LDL-C (adjusted OR = 5.24,95%CI: 2.42–11.34). Poor BP was connected to the elevated TC in older subjects (adjusted OR = 2.48, 95%CI:1.31–4.70). These associations were also strong in subjects < 60 years old. Subjects who reported current and past smoking were associated reduced HDL-C levels without regard to age (age group < 60 years: adjusted OR = 2.79, 95% CI: 2.05–3.80; age group ≥60 years: adjusted OR = 2.96, 95% CI: 1.67–5.25).

## Discussion

This study summarizes the epidemiological characteristics of one minority group within the large-scale CMEC study in China. We found clear evidence that several major health behaviors and metabolic risk factors were associated with dyslipidemia. This was particularly true for metabolic risk factors. These findings have considerable practical relevance for the prevention of dyslipidemia in ethnic minority groups. These factors have been identified as important bio-markers for the presence of dyslipidemia and are also associated with atherosclerotic diseases [18]. Notably, these health behaviors and metabolic factors associated with dyslipidemia can be largely controlled by adopting healthy lifestyle. Dyslipidemia is a part of a larger metabolic syndrome (MS) that puts people at risk for CVD and death, which has long been demonstrated in Framingham and other studies [19, 20]. Therefore, a prevention strategy can be formulated to reduce the prevalence of dyslipidemia, which will then serve to depress CVD.

This is the first study to systematically evaluated the prevalence of dyslipidemia among the ethnic Miao adult population of southwest China. Our analysis describes a dyslipidemia prevalence of 32.8%, which is lower than the national average of 40.4% [17], was also slightly lower than that reported in Guangxi, Jiangsu, Beijing [21–23]. Although formal comparisons have not been made, differences between populations may be related to genetic predisposition, and socioeconomic conditions of the participants [8, 24]. In addition, the prevalence of dyslipidemia components were significantly different. The most common component of dyslipidemia was elevated TG, with prevalence of 21.8% and was consistent with other studies [25, 26]. This phenomenon may reflects the increasing popularity of carbohydrates and high cholesterol diets in recent decades [27].

We found a consistent pattern of increasing likelihood for the presence of dyslipidemia with other indicators of poor cardiovascular health: poor WHR, BMI, BP and FBG. The prevalence of metabolic risk factors are indicative that dyslipidemia is likely to be present. In light of these results, screening for dyslipidemia should be one of the monitoring interventions conducted in the Miao people when obesity, hypertension, or hyperglycemia is observed.

Regardless of age or gender, poor BMI, WHR, FBG and BP results were commonly associated with elevated TG, which corresponds with the results of previous studies [28, 29]. Again, if one metabolic risk factor is detected, other risk factors such as TG should also monitored. Of note, poor WHR was positively associated with TC as well as other lipid components, regardless of gender and age. There is evidence that indicates WHR may well reflect body fat distribution, and the intra-central deposition of adipose tissue is connected to dyslipidemia [30, 31]. In Miao women, FBG is closely associated with elevated TC, LDL-C, TG, and low HDL-C. Recent evidence suggested that lipid levels may possess the ability to stimulate insulin release, which may contribute to higher FBG levels [32].

The present study found that Miao men smoke more than women. Males who are current smoking more likely to have low HDL-C than non-smokers. However, regardless of age stratification, current smokers were more likely to have elevated TG and low HDL-C. These findings were consistent with numerous previous studies and appears to be common across different populations [33, 34]. Prior observations reported that smokers have increased TG and decreased the HDL-C, compared with non-smokers [35]. The impact of smoking on serum lipid levels is also dose-dependent, which may be the core mechanism by which smoking causes atherosclerosis [36]. Furthermore, our study found that drinking alcohol regularly was associated with elevated TG specifically in Miao men, or in subjects younger than 60 years old. Interestingly, this study also revealed that men who are regular drinkers were less likely to have elevated LDL-C than non-drinkers, which was different from the result of the Sun et al. [6] There is evidence to suggest that alcohol consumption can adversely affect the lipid levels. For example, wakabayashi et al. found that heavy drinkers showed detrimental effects on the TG and HDL [37]. A recent study reported that large amounts of alcohol consumption usually increased TG levels. The presence of drinking is a notable issue in atherosclerosis prevention and it might be more important for people with dyslipidemia to avoid alcohol consumption to depress the risk of CVD.

Other researchers observed that the risk of dyslipidemia is increased when physical activity is decreased [38]. However, the current study did not observed a significantly association between physical activity and dyslipidemia. Calculating the metabolic equivalent task of total physical activity based on subject reporting may not be the most precise way to estimate energy use. The present study did not distinguish domain-specific physical activity, thus a future study may more accurately assess the association between these two factors. Shigeki Kinuhata et al. observed a positive correlation between sleep duration and TG abnormality [39]. Zhan et al. also found that sleep duration was significantly associated with dyslipidemia in women, but not in men [40]. However, there was no association of sleep duration with any lipid components in current study. The present analysis focused on night-time sleep rather than on 24-h sleep, and data on sleep duration were collected from questionnaire, but not from objective measures. A meta-analysis also found that sleep duration was linearly associated with increased mortality risk [41]. Sleep quality may be an important factor in mechanisms linking sleep duration and health outcomes [42]. Therefore, further studies will be needed to investigate the association of sleep duration with health status.

Several limitations are present in this study. This analysis adjusted for factors that may confound the outcome, but residual confounding factors are possible. Behavioral factors such as smoking and alcohol use were self-reported, which may lead to misclassification. We conducted a cross-sectional study that cannot determine causation. Although we found that certain characteristics appeared together statistically, we could not determine which factor preceded another. A study using longitudinal follow-up data would be needed to assess the risk of an outcome. Nevertheless, this is the first known study in the Miao minority ethnic group to quantify behavioral, clinical, and metabolic factors and their relationship with dyslipidemia. This has considerable practical implications, findings from the present study have shed light on the cost-effective strategy for dyslipidemia management based on lifestyle changes in ethnic Chinese minority groups.

## Conclusions

This large-scare study of Miao Chinese adults provided convincing evidence that several modifiable risk factors for CVD were closely linked with the prevalence of dyslipidemia. These findings may be used to inform the development of population-based CVD prevention strategies. Given the limited economic and medical conditions in regions with large ethnic minorities, such interventions are a cost-effective way to respond to the challenges posed by dyslipidemia and even CVD.

## Data Availability

Currently, the database used to support this study are not freely available in view of participants’ privacy protection but are available from the corresponding author on reasonable data request. Researchers interested in our study could contact the corresponding author Dr. Feng Hong (519490967@qq.com) who will review the data request.
